# Attenuation of adipose tissue inflammation by pro-resolving lipid mediators

**DOI:** 10.1016/j.coemr.2024.100539

**Published:** 2024-09

**Authors:** Madison Clark, Bianca E. Suur, Matúš Soták, Emma Börgeson

**Affiliations:** 1 Department of Biomedicine, Aarhus University, Aarhus, Denmark; 2 Steno Diabetes Center Aarhus, Aarhus University Hospital, Aarhus, Denmark; 3 Department of Clinical Immunology and Transfusion Medicine, Sahlgrenska University Hospital, Gothenburg, Sweden

## Abstract

Adipose tissue inflammation drives systemic pathophysiology, for instance, obesity-related cardiometabolic disease. Specialized pro-resolving lipid mediators are a superfamily of endogenously produced lipids that promote the resolution of inflammation, an actively regulated process. New evidence suggests that such lipids (e.g. lipoxins) could resolve adipose tissue inflammation and, thus, subvert obesity-related diseases. A key feature of pro-resolving lipids is their ability to promote an M2-like macrophage phenotype and enhance efferocytosis while avoiding adverse side-effects typically associated with anti-inflammatory drugs, such as increased sensitivity to infections. This brief review discusses the therapeutic potential of pro-resolving lipid mediators in mitigating systemic disease fueled by adipose tissue inflammation in both experimental and human disease models.

## Innovative approaches to tackle adipose tissue inflammation

Adipose tissue inflammation is a form of metaflammation strongly linked to the development of metabolic and cardiovascular disease [1, 2, 3, 4]. While characterized by low-grade chronic inflammation and the presence of proinflammatory adipose tissue macrophages, it does not coincide with localized heat or pain, which differentiates it from classic inflammation. An inflammatory state disrupts the normal physiological functions of adipose tissue and contributes to systemic inflammation as well as pathophysiology in other organs [5,6]. Both preclinical and clinical research have confirmed the paradigm that adipose tissue inflammation is mechanistically linked to metabolic disease, and reducing metaflammation has therefore been proposed as a therapeutic strategy to subdue the disease [4]. Indeed, this approach improves systemic insulin sensitivity and lowers the risk of developing obesity-related complications. Nonetheless, conflicting results have been reported, likely due to the intricately intertwined cross-talk of concomitant proinflammatory and anti-inflammatory signaling cascades [7].

The primary function of white adipose tissue is energy storage and release, and the efficient switching between these states, in accordance with physiological needs, is referred to as metabolic flexibility. Indeed, a hallmark of metabolic diseases and adipose tissue dysfunction is metabolic inflexibility, which also has a central role in immune metabolism and metaflammation [8]. Adipose tissue is also a vital endocrine organ that secretes adipokines, hormones, and other signaling molecules that regulate various physiological processes, including metabolism, appetite, inflammation, and immune function [9]. While consisting primarily of adipocytes, adipose tissue also contains various other cell types, such as preadipocytes, fibroblasts, endothelial cells, and notably, immune cells. The tissue is structurally supported by an intricate extracellular matrix, and in the case of obesity-induced adipocyte hypertrophy, this network must be degraded and rebuilt adequately to allow tissue expansion without the induction of adipose tissue inflammation [10,11]. Importantly, adipose tissue is heavily vascularized, and endothelial cells have a critical role in regulating the initiation of adipose tissue inflammation, as recently reviewed [12]. Furthermore, obesity-related inflammation may also affect the sympathetic nerve activation and thus lipolysis [13]. Although the root cause of adipose tissue inflammation is debated, the tissue-resident immune cells, such as macrophages, have a critical role. Activated immune cells amplify the inflammatory response by releasing additional proinflammatory mediators and promoting adipose tissue dysfunction. This results in a self-amplifying negative feed-forward cycle that culminates in pathophysiological conditions, such as cardiometabolic disease [7].

Many have proposed, ourselves included, utilizing pro-resolving lipids, such as lipoxins, as a potential therapeutic approach to resolve adipose tissue inflammation and subvert obesity-related disease [14, 15••, 16••]. As reviewed below, a key feature of pro-resolving lipids is their ability to promote an M2-like macrophage phenotype and enhance efferocytosis, which is critical for successful inflammatory resolution [17]. Importantly, pro-resolving lipids appear to largely avoid adverse side-effects typically associated with anti-inflammatory drugs, such as increased sensitivity to infections [18, 19, 20]. Here, we review these novel approaches to tackling adipose tissue inflammation that fuels systemic diseases.

## Specialized pro-resolving lipid mediators

**Figure 1:**
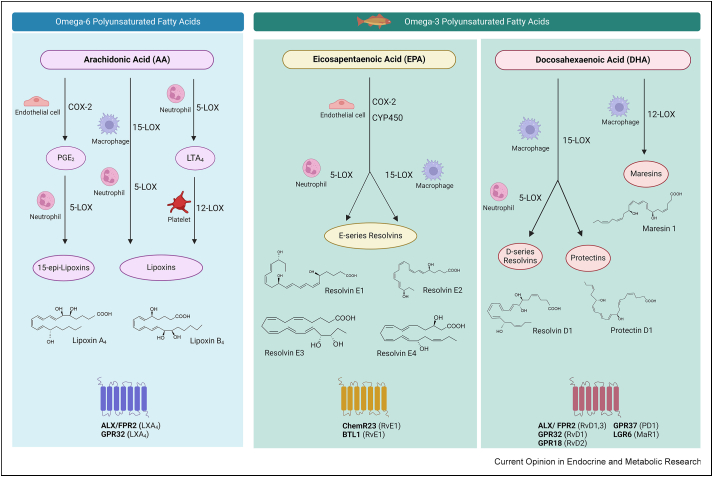
**Overview of the specialized pro-resolving mediator synthesis pathways**. The figure provides a schematic overview of the biosynthesis of specialized pro-resolving lipid mediators (SPMs). ω-6 polyunsaturated fatty acids (PUFAs), specifically arachidonic acid (AA), can be converted into lipoxin A_4_ (LXA_4_) and lipoxin B_4_ (LXB_4_). This is achieved through the sequential actions of lipoxygenase (LOX) and cyclooxygenase (COX) enzymes, as depicted in the figure. While the LXB_4_ receptor remains unidentified, LXA_4_ binds the formyl peptide receptor 2 (ALX/FPR2) and G-protein-coupled receptor 32 (GPR32). In a similar manner, the ω-3 PUFAs, specifically eicosapentaenoic acid (EPA) and docosahexaenoic acid (DHA) can be converted into resolvins, protectins, and maresins. EPA- and DHA-derived SPMs signal through the ALX/FPR2, GPR32, GPR18, ChemR23/CMKLR1, BTL1, LGR6, and GPR37 receptors.

Derived from polyunsaturated fatty acids (PUFAs), specialized pro-resolving mediators (SPMs) are a superfamily of bioactive lipids that promote the resolution phase of an inflammatory reaction, a process once thought to be a passive occurrence, but which is currently recognized as being actively regulated [21,22]. The principal source of endogenous SPMs are immune and endothelial cells. SPMs are enzymatically derived from two separate polyunsaturated fatty-acid classes: ω-6 and ω-3 fatty acids (Figure 1); their biosynthesis pathways have been extensively reviewed elsewhere [23]. SPMs with pro-resolving properties include bioactive lipids of the lipoxin, resolvin, protectin, and maresin families, all of which facilitate the resolution of inflammation by decreasing neutrophil recruitment. Simultaneously, they support a favorable M2-like macrophage activation, enhance efferocytosis, and shift cytokine production toward a resolving state. This involves elevating levels of TGF-β and IL-10 while diminishing pro-inflammatory cytokines, as comprehensively discussed in a recent review [24]. These mediators thus act as endogenous signals to actively resolve inflammation, thereby preventing its chronicity and associated tissue damage [25].

**Figure 2:**
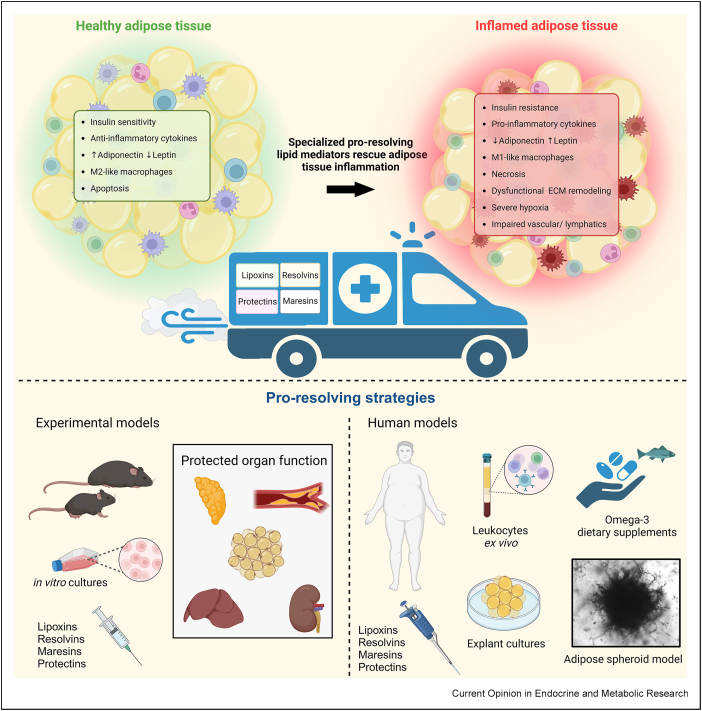
**Hallmarks of obesity-induced adipose tissue inflammation and pro-resolving strategies under investigation to address obesity-related disease burdens.** The top section of the figure provides a schematic overview of the hallmarks of adipose tissue in a healthy or inflamed state. Specialized pro-resolving mediators (SPMs) can initiate inflammatory resolution, thus enabling restoration of tissue homeostasis. The lower section of the figure illustrates pro-resolving strategies, including administration of lipoxins, resolvins, maresins, and protectins. Various experimental models (lower-left panel) have been used to demonstrate SPM-mediated protection from obesity-induced organ injury, e.g. restored glucose tolerance, blood vessels, adipose tissue, liver, and kidney. Furthermore, a variety of human models (lower-right panel) have been used to evaluate the mechanisms through which pro-resolving strategies function to modulate the inflammatory response in obesity.

Lipoxins are a family of lipid mediators comprising two members: lipoxin A_4_ (LXA_4_) and lipoxin B_4_ (LXB_4_). Lipoxins are formed from arachidonic acid, an ω-6 fatty acid found in the phospholipids of cell membranes, through a series of enzymatic reactions involving lipoxygenases (LOXs) (Figure 1). Intriguingly, low-dose aspirin induces the formation of aspirin-triggered lipoxins 15-epi-LXA_4_ and 15-epi-LXB_4_ through acetylation of COX2, and epi-lipoxins have a more stable conformation, resulting in a decreased degradation, compared to LXA_4_ and LXB_4_, and enhanced pro-resolving effects [26]. Lipoxins play a pivotal role in the resolution phase of inflammation and have repeatedly been shown to reduce adipose tissue inflammation [27]. Furthermore, lipoxin treatment has been shown to specifically attenuate disease progression *in vivo* in a range of disorders, including atherosclerosis and cardiometabolic disease, as discussed in the following (Figure 2) [28,29].

Resolvins, maresins, and protectins are SPMs that originate from ω-3 PUFAs, such as eicosapentaenoic acid (EPA) and docosahexaenoic acid (DHA) (Figure 1). The resolvin family is composed of D-series resolvins (RvD1-RvD6) derived from DHA and E-series resolvins (RvE1-RvE4) derived from EPA. The protectins and maresins families are less extensive, and protectins (PD1) and maresins (MaR1 and MaR2) are derived from DHA [30]. Similar to lipoxins, these three classes of mediators hold potential as an intriguing therapeutic strategy for mitigating inflammation [24].

In contrast to ω-6 fatty acids, ω-3 fatty acids are not naturally synthesized within mammals. Consequently, the sole means of acquiring them is through dietary sources. An interesting line of research involves studies that unravel ways to convert ω-6 to ω-3, for instance, by genetically modifying farm animals to carry the *Fat-1* gene. In mice, carriers of the *Fat-1* gene can convert ω-6 fatty acids to ω-3 fatty acids. This strategy could enhance the ω-3 fatty acid content of constituents commonly used for human consumption [31]. Furthermore, this genetic mutation protected carrier mice from high-fat, diet-induced, nonalcoholic fatty liver disease, concomitant with an attenuated inflammatory profile in the brown adipose tissue, and improved glucose tolerance [32].

## Resolving adipose tissue inflammation by harnessing the power of pro-resolving lipid mediators

Lipoxins have been shown to attenuate obesity-related adipose tissue inflammation in patients with low-grade systemic inflammation using *ex vivo* culture systems (Figure 2) [16]. First, it was shown that three-dimensional cell culture systems amplify lipoxin-mediated responses in cultured adipocytes [16] when using human cells [16,33,34]. Secondly, lipoxins reduced obesity-related inflammation in human omental adipose tissue explants obtained from obese gastric bypass patients. However, the effect appeared specific to a subgroup of responder patients. Interestingly, although all obese patients had similar systemic levels of inflammatory cytokines (e.g., IL-8 and IL-6) compared to the age- and sex-matched lean control group, the lipoxin-responder group had higher plasma levels of C-reactive protein (CRP); this hepatic acute-phase protein is often used as an inflammatory marker. The study also showed that CRP can elicit an inflammatory response in cultured adipocytes. The lipoxin-responders appeared more prone to produce anti-inflammatory oxylipins and to stimulate an anti-inflammatory M2-like macrophage phenotype [16]. Furthermore, the responders displayed elevated prostaglandin D2 levels [16], which has been interlinked with transcription of lipoxin-generating enzymes [35].

Lipoxin-treatment may require a personalized medicine approach to be effective, as shown in other human studies where lipoxins modulate neutrophil oxidative burst, integrin expression, and lymphatic transmigration differentially in human health and atherosclerosis [15]. One of the key findings in this study was that lipoxins increased the oxidative burst in neutrophils from healthy controls, indicating that pro-resolving lipids may aid neutrophil function in health. Conversely, in neutrophils from patients with atherosclerosis, lipoxins reduced the excessive reactive oxygen species (ROS) production associated with cardiovascular disease. Importantly, in the patient-specific cells, lipoxins also downregulated activation of CD11b integrin, which has a central role in clot activation and promoted neutrophil transmigration through lymphatic epithelial monolayer [15]. The latter mechanism may be a crucial mechanistic clue to how lipoxins promote the resolution of inflammation as the lymphatics have a central role in transporting leukocytes away from the site of inflammation [36].

In addition to the evidence in human *ex vivo* studies, animal studies have demonstrated that lipoxin-mediated protection against adipose tissue inflammation is mediated by a switch of the adipose tissue–residing macrophage phenotype from a proinflammatory M1-like state to a resolving M2-like state. This is in turn coupled to attenuation of adipocyte TNF-α secretion [28,37]. Another therapeutic approach could be to increase the endogenous production of lipoxins through modulation of the enzymes responsible for the metabolism of arachidonic acid toward lipoxin production. This may be challenging as the LOX enzymes are also critically involved in the synthesizing proinflammatory leukotrienes and prostaglandins. However, some *in vitro* studies using cocultures of mesenchymal stem cells and peripheral blood mononuclear cells have shown that overexpression of 15-LOX in mesenchymal stem cells shifts the Th17/Treg ratio toward the pro-resolving Treg state, which is interestingly correlated with increased LXA_4_ production [38]. Another possibility would be to alter the intracellular localization of 5-LOX, as nuclear 5-LOX favors the biosynthesis of proinflammatory leukotriene B4, whereas, in theory, cytoplasmic 5-LOX could favor the biosynthesis of lipoxins [39].

Despite the ever-growing body of evidence showing that marine-derived ω-3 polyunsaturated fatty-acid supplementation results in health benefits, the effects of ω-3 supplementation on adipose tissue inflammation have yielded inconclusive results. In a recent double-blind, randomized trial, fish-oil concentrate supplementation for 12 weeks was compared to a placebo control receiving corn oil [40]. In obese participants, the dietary ω-3 PUFAs modulated the adipose expression of inflammatory genes but had limited effect on the ω-3-derived SPM levels [40]. Interestingly, the effects of the ω-3 PUFA supplementation was more pronounced in the lean cohort [40]. Furthermore, another study using a short-term, 4-week intervention in obese women scheduled for bariatric surgery, compared to consuming a 2-week low-calorie diet, showed a reduction in some inflammatory markers in adipose tissue, such as *CD45* and *CCL2* [41].

Interestingly, a modest weight loss in patients with metabolic syndrome increased their neutrophils' propensity to secrete RvE1 upon stimulation [42]. In a murine study using Diversity Outbred mice to mimic human obesity, RvE1 treatment after a high-fat diet showed that 50% of the mice were SPM responders, where improvements in gastric inhibitory peptide, insulin, glucagon, and leptin levels were observed. In the group of mice with lower body-fat mass, RvE1 treatment improved hyperleptinemia [43]. Taken together, the data from human and murine studies indicate a therapeutic window for RvE1 that needs to be considered for future clinical trials involving patients with varying levels of obesity.

Another member of the SPM family is RvD1, which has been investigated in adipose tissue inflammation. In a coculture system of human adipocytes and human macrophages; RvD1 reduced proinflammatory cytokine secretion in a dose-dependent manner through modulation of the macrophages as opposed to through direct effects on the adipocytes [44]. Furthermore, RvD1 treatment reduced adhesion and transmigration of macrophages in a human *in vitro* assay [45]. Additionally, RvD1 skewed macrophage phenotype from an M1-like state toward the pro-resolving M2-like state and enhanced the phagocytic capacity of the cells [46].

The effects of MaR1 on glucose uptake and insulin signaling have been examined in human subcutaneous adipocytes from overweight and obese individuals. MaR1 modulated the inhibitory effects of TNF-α on insulin-stimulated glucose uptake and Akt phosphorylation in adipocytes. The impact of MaR1 has also been shown in experimental animal models. In control mice, MaR1 treatment for 3 h induced Akt phosphorylation in white adipose tissue and skeletal muscle but did not enhance the effects of insulin [47]. Conversely, a 10-day treatment regimen of MaR1 administration in diet-induced obese mice reduced hyperglycemia, improved insulin sensitivity, and partially restored impaired insulin response in skeletal muscle [47]. These findings imply that MaR1 has the potential to alleviate the disruption of insulin signaling caused by obesity.

Maresins have also been shown to promote resolution by activating brown adipose tissue, which is an adipose depot specialized for energy expenditure and plays a pivotal role in adaptive thermogenesis. Sugimoto et al. recently revealed a link between cold exposure and β3-adrenergic stimulation, where brown adipose tissue produces MaR2 to aid the inflammatory resolution process. Following production, MaR2 acts on macrophages in the liver by promoting the phagocytic functions of the Kupffer cells [48]. This finding was similar to another study, where MaR1 promotes brown adipose tissue activation and browning/beiging of white adipose tissue through thermoregulation of adipocytes, polarization of macrophages toward an M2-like phenotype, and increased expression of anti-inflammatory macrophage markers [49].

## Conclusion and perspectives

Inflammation is an evolutionarily conserved process fundamental to everyday life. However, chronic inflammation has been linked to a wide variety of diseases [3]. Adipose tissue inflammation is an essential part of this puzzle as a growing body of evidence shows that inflammation within the fat pads is a key driver of obesity-related pathophysiology. Here, we have reviewed evidence that supports the therapeutic potential of pro-resolving lipid mediators in mitigating systemic disease fueled by adipose tissue inflammation. A particularly tempting characteristic of these compounds is their ability to promote inflammatory resolution while avoiding adverse side-effects typically associated with anti-inflammatory drugs, such as increased sensitivity to infections. In animals, the impact of pro-resolving lipids is well established, and the lipid-mediated reduction of inflammation in the fat pads is substantial enough to reduce obesity-related systemic disease. Human studies have not come as far but are mainly supported by *ex vivo* treatment of cells and adipose tissue explants; although the results from this work appear promising, the data also indicate that a personalized medicine approach may be needed to harness the full power of pro-resolving therapeutics in humans. More studies are needed to clarify what this entails fully and to uncover the true therapeutic value of pro-resolving lipids, the field must move toward more elaborate human studies and trials.

## Author contributions

All authors contributed conceptually and with both writing and editing of the manuscript. MC designed the figures, with input from all authors. All authors read and approved the final version of the paper.

## Declaration of competing interest

The authors declare that they have no known competing financial interests or personal relationships that could have appeared to influence the work reported in this paper.

## Data Availability

No data were used for the research described in the article.
